# Phylogenetic Analysis and Expression Patterns of Triterpenoid Saponin Biosynthesis Genes in 19 Araliaceae Plants

**DOI:** 10.3390/ijms26073439

**Published:** 2025-04-07

**Authors:** Chi Ma, Yu Lin, Junjun Yin, Lijuan Zhu, Jinkai Fang, Dan Zhang

**Affiliations:** School of Tropical Agriculture and Forestry, Hainan University, Haikou 570228, China; mc1416144603@163.com (C.M.); 15008972818@163.com (Y.L.); 17680262946@163.com (J.Y.); z18314437507@163.com (L.Z.); fangjinkai1997@163.com (J.F.)

**Keywords:** Araliaceae, transcriptome, phylogenetics, species tree, ginsenosides

## Abstract

The Araliaceae family has significant economic and medicinal value. However, the phylogenetic relationships and the expression patterns of key genes of the active triterpenoid substance within this family are still unclear. In this study, we employed comparative transcriptomics to analyze the transcriptomes of 19 species from 11 genera of Araliaceae, aiming to elucidate the evolutionary history of the family and the expression patterns of key genes in the ginsenoside biosynthesis pathway. Our results divide Araliaceae into two subfamilies: Aralioideae and Hydrocotyloideae. Aralioideae is further classified into three groups: the *Aralia–Panax* group, the *Polyscias–Pseudopanax* group, and the Asian Palmate group. PhyloNet analysis reveals that the common ancestor of *Panax ginseng*, *Panax quinquefolius*, and *Panax japonicus* was an allopolyploid, likely resulting from hybridization between *Panax notoginseng* and *Panax pseudoginseng*. Additionally, all Aralioideae species underwent the pg-β event, which may be critical for ginsenoside biosynthesis. We discovered that *Panax* species exhibit distinct expression patterns of key enzyme genes (*β-AS*, *DDS*, *CYP450*, *UGTs*) compared to other Araliaceae species. These enzyme genes show independent evolutionary lineages in gene trees, suggesting unique functional adaptations that enable *Panax* species to efficiently synthesize ginsenosides. This study provides a theoretical foundation for the conservation and utilization of Araliaceae germplasm resources.

## 1. Introduction

The Araliaceae family comprises approximately 45 genera and over 1500 species, widely distributed across tropical and temperate regions of both hemispheres. These plants exhibit rich morphological diversity, including trees, shrubs, and perennial herbs [1]. The family Araliaceae is divided into two subfamilies: Aralioideae and Hydrocotyloideae [2]. Many species in the Aralioideae subfamily possess medicinal value, with the most renowned being *Panax* species such as *P. ginseng*, *P. notoginseng*, and *P. quinquefolius*. In China, the dried roots of *P. ginseng* and *P. notoginseng* have been used as medicinal herbs for thousands of years, as documented in numerous classical medical texts, such as Shen-nong’s *Herbal Classics* and *Compendium of Materia Medica* [3,4]. *P. quinquefolius*, native to North America, was later introduced to China and was first recorded in the *Supplement to the Illustrated New Compilation of Materia Medica* in 1694 [5]. Today, these plants are widely utilized not only as medicinal herbs but also as dietary supplements and functional foods on a global scale [6]. Pharmacological studies have revealed that their primary bioactive components, ginsenosides, exhibit diverse biological activities, including cardioprotective, anti-inflammatory, anticancer, and antidiabetic effects [7].

Ginsenosides, classified as triterpenoid compounds, are categorized into dammarane-type and oleanane-type based on their skeleton structure. Among these, dammarane-type ginsenosides are the primary constituents and can be further divided into protopanaxadiol, protopanaxatriol, and ocotillol types [6]. The biosynthetic pathway of ginsenosides originates from the mevalonate (MVA) pathway and the plastid-derived 2-C-methyl-D-erythritol 4-phosphate (MEP) pathway, which synthesize isopentenyl pyrophosphate (IPP) and its isomer dimethylallyl pyrophosphate (DMAPP) [8]. Subsequently, IPP and DMAPP are converted into 2,3-oxidosqualene, which undergoes cyclization, hydroxylation, and glycosylation to ultimately produce ginsenosides [6]. *Panax* species, renowned for their abundant ginsenosides, have been overharvested. Coupled with their long growth cycles and stringent environmental requirements, this has resulted in wild *P. ginseng* becoming endangered and *P. notoginseng* being extinct in the wild [9,10]. Therefore, elucidating the phylogenetic relationships of Araliaceae plants and deciphering the biosynthetic pathway of ginsenosides not only provides a scientific basis for the conservation and sustainable utilization of endangered plants but also offers crucial genetic information for the field of ginsenoside biosynthesis and metabolic engineering. These efforts are of significant importance for preserving biodiversity and enhancing the medicinal value of Araliaceae plants.

The morphological characteristics of Araliaceae plants are highly complex and diverse, with relatively conserved floral traits. As a result, early morphological classifications predominantly relied on floral characteristics. For example, Harms (1898) [11] classified Araliaceae plants into three groups: Aralieae, Mackinlayeae, and Schefflereae, based on the form of the corolla folding. With the advancement of molecular biology, the internal transcribed spacer (ITS) of nuclear rRNA genes and the trnL-trnF region of the chloroplast genome have become critical tools for studying the phylogeny of Araliaceae. These studies indicate that the subfamily Aralioideae can be further divided into four groups: the *Aralia–Panax* group and Asian Palmate group, distributed in East Asia and Southeast Asia, and the *Polyscias–Pseudopanax* group and *Greater Raukaua* group, found in the Pacific and Indian Oceans [1,12,13]. However, studies based on limited genetic fragments provide insufficient phylogenetic resolution, leaving the basal evolutionary relationships unclear. Additionally, the complexity introduced by multiple hybridization events, polyploidization, and rapid evolution presents ongoing challenges for further elucidation of the phylogenetic relationships within Araliaceae.

With the rapid advancements in sequencing technologies and the field of phylogenetics, approaches such as whole-chloroplast genome sequencing, target gene capture sequencing, and reduced-representation genome sequencing have been applied to phylogenetic studies of Araliaceae [2,14,15]. Compared to these methods, transcriptome sequencing has emerged as an essential tool for phylogenetic research due to its richness in informative loci, low cost, and high feasibility [16,17,18]. Single-copy orthologous genes obtained from transcriptomes have demonstrated strong capabilities in resolving complex phylogenetic problems across various taxa. For example, this approach was utilized in the “One Thousand Plant Transcriptomes Project” to reveal the evolutionary relationships of green plants [17]. Additionally, it has been applied in phylogenetic studies of Leguminosae [19], Asteraceae [20], and *Camellia* [16]. However, its application in the Araliaceae family remains relatively limited. Moreover, transcriptome data can provide critical insights into important biological processes such as polyploidization, hybridization, and incomplete lineage sorting [17,21,22]. Since transcriptomes contain gene expression information, they also enable the investigation of expression patterns of key genes involved in the ginsenoside biosynthesis pathway.

With the ongoing progress of sequencing technology, a vast amount of plant transcriptome data has been made publicly available, offering a robust data foundation for plant phylogenetic analysis. We have gathered all available transcriptome data for Araliaceae plants from public databases and successfully obtained transcriptome data from 19 Araliaceae plant species. This study aims to utilize comparative transcriptomics to analyze transcriptome data from 19 publicly available species of the Araliaceae family and two outgroup species from the Apiaceae family (*Centella asiatica* and *Daucus carota*), totaling 21 species. The objectives are to explore the phylogenetic relationships, complex evolutionary history of Araliaceae, and the expression patterns of key genes involved in the ginsenoside biosynthesis pathway. The findings will provide a scientific basis and data foundation for the conservation and sustainable utilization of Araliaceae genetic resources, as well as for the genetic breeding and varietal improvement of economically important species.

## 2. Results

### 2.1. Assembly and Characterization of Transcriptome of Araliaceae Plants

Transcriptomics provides rich genetic information and serves as a crucial data foundation for unraveling the evolutionary history of Araliaceae plants. We collected transcriptome data for 19 species of Araliaceae plants from public databases and selected *C. asiatica* and *D. carota* from the Apiaceae family as outgroup species, utilizing transcriptome data from a total of 21 species. De novo transcriptome assembly was performed using Trinity v2.15.1, yielding 6,098,021 transcripts with an N50 value of 1467 bp (Table S1). The completeness of the assembled transcripts was assessed using BUSCO v5.4.7 with the core eudicot database. The results showed that the transcriptome assemblies for most species had completeness levels exceeding 90%, except for *Hedera helix* (54.10%) and *Hydrocotyle umbellata* (54.40%), which had completeness levels below 80% (Table S2). The lower completeness in these two species is likely due to the use of transcriptome sequencing data from only a single tissue and the lack of replicates. Overall, the high completeness of our transcriptome assemblies provides a robust foundation for subsequent analyses.

Functional annotation of the predicted proteins was performed using seven databases: SwissProt, PFAM, KEGG, GO, COG, TAIR11, and Non-Redundant Protein Database (NR). A total of 5,023,301 protein sequences were successfully annotated with known proteins or domains (Table S3). Transcription factor (TF) annotation conducted using ITAK v1.6 identified 59208 protein sequences belonging to TF families, with an average of 2819 per species. Among these, AP2/ERF-ERF (*n* = 4137) was the most abundant TF family in the Araliaceae transcriptomes, followed by C2H2 (*n* = 4060) and bHLH (*n* = 4030) (Table S4). Additionally, 79,585 simple sequence repeat (SSR) loci were identified (Table S5). Of these, 87 loci exhibited polymorphism between individual plants, providing valuable genetic resources for genomic studies and breeding efforts in Araliaceae species (Table S6).

### 2.2. Phylogenetic Trees and Divergence Times of Araliaceae Plants Based on Comparative Transcriptomic Analysis

**Figure 1:**
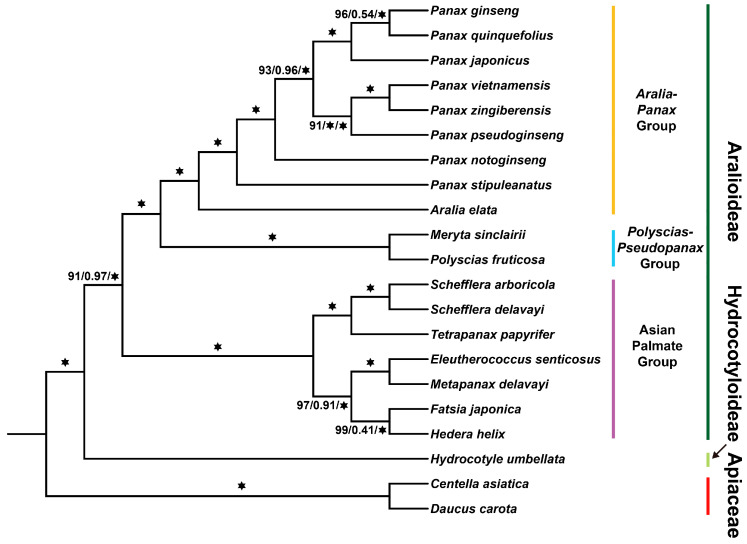
Phylogenetic relationships of Araliaceae plants based on 510 single-copy orthologous genes. The concatenation-based method uses IQTREE and MrBayes for tree construction, while the coalescent-based method employs ASTRAL for tree building. The three phylogenetic trees have the same topological structure. The nodes with the highest support in the three trees are indicated by “
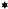
”. Otherwise, the support values are indicated in the format of IQTREE (bootstrap support)/ASTRAL (local posterior probability)/MrBayes (posterior probability). The subfamilies and groups to which the Araliaceae plants belong are marked on the right side.

Obtaining accurate single-copy orthologous genes from the transcriptome data will enable us to correctly resolve the phylogenetic relationships among Araliaceae plants. We identified 510 single-copy orthologous genes across the 21 species. We used these data, combined with both concatenation-based and coalescent-based methods, to construct a phylogenetic tree in order to reveal the evolutionary relationships and taxonomic status within the Araliaceae family. In the concatenation-based method, we constructed a maximum likelihood tree using IQ-TREE v2.2.5 and a Bayesian tree using MrBayes v3.2.7a. The tree in the coalescent-based method was constructed using ASTRAL v5.7.8. The three species trees we ultimately obtained consistently displayed the same phylogenetic relationships (Figure 1). Only the concatenation-based tree showed relatively low branch support for *P. ginseng* and *P. quinquefolius* (local posterior probability = 0.54), as well as for *Fatsia japonica* and *H. helix* (local posterior probability = 0.41). All other branches showed high support [IQ-TREE bootstrap support > 90, MrBayes posterior probability > 0.9, ASTRAL local posterior probability > 0.9], with most branches receiving maximum support, indicating that the reliability of our phylogenetic trees is very high (Figure 1). The species tree showed that the Araliaceae family is divided into two major subfamilies: Hydrocotyloideae, represented by *Hydrocotyle* species, and Aralioideae, which includes other Araliaceae plants. Within Aralioideae, the Asian Palmate group diverged first, forming the sister clade to the remaining Aralioideae species. The *Polyscias–Pseudopanax* group and the *Aralia–Panax* group, which includes the *Panax*, are placed within the same major clade, where they are sister branches to one another (Figure 1).

**Figure 2:**
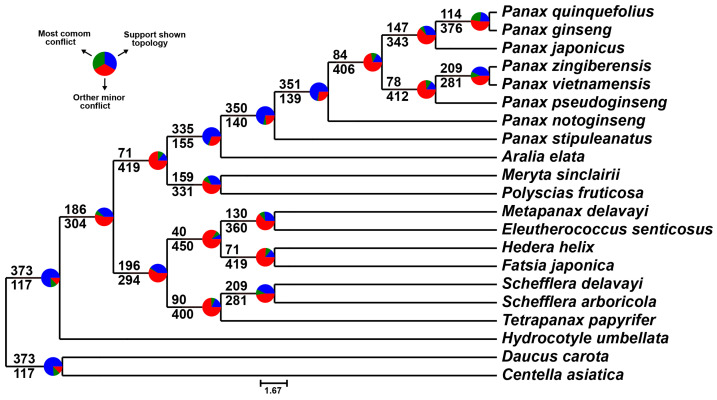
In the species tree constructed by ASTRAL, PhyParts is used to infer conflicts between the species tree and gene trees. Pie charts represent the gene trees inferred by PhyParts, showing the proportion of gene trees that are concordant with the species tree (blue), represent the most common alternative topology (green), and other alternative topologies (red).

Due to the lower support for the branches of *P. ginseng* and *P. quinquefolius* (local posterior probability = 0.54), as well as *F. japonica* and *H. helix* (local posterior probability = 0.41) in the coalescent-based tree, we used PhyParts v0.0.1 to detect conflicts between the species tree and gene trees in order to investigate the reasons for the lower support of these two branches. In the PhyParts v0.0.1 results, the sister relationship between *P. ginseng* and *P. quinquefolius* was supported by 114 gene trees, accounting for 23.3% of all gene trees, while the most consistent conflicting gene trees accounted for 24.1% (Figure 2). The sister relationship between *F. japonica* and *H. helix* was supported by 71 gene trees, representing 14.5% of all gene trees, with the most consistent conflicting gene trees accounting for 10.6% (Figure 2). These findings suggest that the low branch support may be attributed to incomplete lineage sorting (ILS) or hybridization. Nevertheless, our three species trees share the same topology, and the concatenation-based maximum likelihood and Bayesian tree analyses provided high support for these two branches [bootstrap support = 96 and 99, posterior probability = 1] (Figure 1). This indicates that phylogenetic analyses using larger datasets can mitigate the impact of incomplete lineage sorting on the topology, thereby yielding more accurate phylogenetic relationships. In conclusion, the phylogenetic tree of Araliaceae constructed based on 510 single-copy orthologous genes demonstrated high robustness. These results suggest that identifying and utilizing a sufficient number of single-copy orthologous genes from the transcriptomes of Araliaceae plants can effectively reveal accurate phylogenetic relationships.

**Figure 3:**
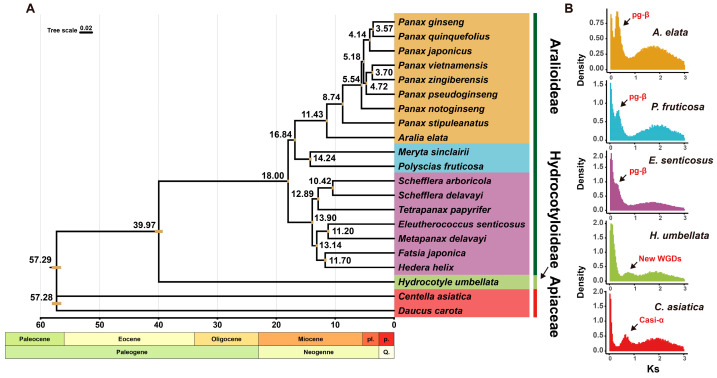
Estimation of divergence time and Whole-Genome Duplication (WGD) events in Araliaceae plants. (**A**) Divergence time estimation. The numbers at the nodes represent the average divergence time, and the light brown bars indicate the 95% range of the highest posterior density (HPD) interval for divergence time. A geological time scale is shown at the bottom. The star indicates the pg-β event. (**B**) WGD events inferred from the *Ks* distribution of homologous genes in four Araliaceae plants (*Aralia elata*, *Polyscias fruticosa*, *Eleutherococcus senticosus*, *H. umbellata*) and *C. asiatica* from Apiaceae. The color of each species’ *Ks* plot matches the color of the group to which it belongs.

Based on the reliable phylogenetic topology of Araliaceae, we estimated the divergence times within the family. The results indicate that Araliaceae originated approximately 57.29 million years ago (MYA) [95% HPD = 56.5, 58.12]. The divergence between Hydrocotyloideae and Aralioideae occurred around 39.97 MYA [95% HPD = 39.29, 40.64]. The diversification of Aralioideae began approximately 18 MYA [95% HPD = 17.85, 18.16]. The emergence of *Panax* dates back to around 11.43 MYA [95% HPD = 11.24, 11.62], while the origin of the tetraploid lineage within *Panax* (including *P. ginseng*, *P. quinquefolius*, and *P. japonicus*) is estimated to have occurred approximately 5.18 MYA [95% HPD = 5.07, 5.28] (Figure 3A).

### 2.3. The Evolutionary Origin of Polyploid Panax Species

**Figure 4:**
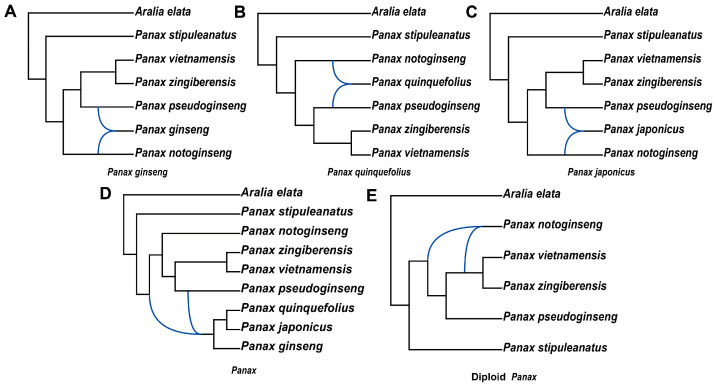
Phylogenetic network of *Panax*. (**A**–**C**) Using the InferNetwork_MP_Allopp method in PhyloNet, phylogenetic networks were inferred by setting each polyploid species as a hybrid species. (**D**) Using the InferNetwork_MP_Allopp method in PhyloNet, a phylogenetic network was inferred by setting three polyploid species as hybrid species. (**E**) Using the InferNetwork_MPL method in PhyloNet, a phylogenetic network was inferred for diploid *Panax* plants. Blue branches indicate lineages involved in reticulate history.

*P. ginseng* in the Araliaceae family has been confirmed as an allopolyploid species in previous studies, and both its close relatives, *P. quinquefolius* and *P. notoginseng*, are also considered allopolyploid species. However, the lack of research on their ancestral progenitors has resulted in gaps in the understanding of this segment of their evolutionary history. Our transcriptome data includes several well-known species of *Panax* (eight species), which enables us to explore the origin of the parental species of the allopolyploid plants. We used the “InferNetwork_MP_Allopp” method in PhyloNet v3.8.2 to infer phylogenetic networks for the three tetraploid species within *Panax* based on gene trees. In the analyses of *P. ginseng*, *P. quinquefolius*, and *P. japonicus*, the results from PhyloNet v3.8.2 indicated that they share the same putative parental species: *P. notoginseng* and *P. pseudoginseng* (Figure 4A–C). In the phylogenetic network of *Panax* species, the three tetraploid species were positioned within the same branch, with their common ancestor identified as an allopolyploid (Figure 4D). This suggests that *P. quinquefolius* and *P. japonicus* may also be allopolyploid species, and their ancestors likely underwent an allopolyploidization event in the Southeast Asian region, where diploid species of the *Panax* are primarily distributed. However, the putative parental ancestors differed between the two analyses. In the phylogenetic network of *Panax*, the common ancestor of *P. pseudoginseng*, *P. notoginseng*, *Panax vietnamensis*, and *Panax zingiberensis* was found to hybridize with *P. pseudoginseng* (Figure 4D). As a result, we could not definitively determine the parental ancestors of the shared common ancestor of the three tetraploid species. To further investigate the causes of these discrepancies, we employed the “InferNetwork_MPL” method in PhyloNet v3.8.2 to construct a phylogenetic network for diploid species within *Panax*. The results revealed that *P. notoginseng* is a hybrid species, with the common ancestor of *P. vietnamensis*, *P. zingiberensis*, and *P. pseudoginseng* hybridizing with the common ancestor of *P. vietnamensis* and *P. zingiberensis* (Figure 4E). We speculate that the hybridization events occurring in the ancestors of diploid *Panax* species resulted in extensive gene flow, making it challenging to determine the parental ancestors of the allopolyploid species. By combining the phylogenetic networks of the allopolyploid species and the *Panax*, we found that in all four results, *P. pseudoginseng* is a hybrid species. This phenomenon suggests that *P. pseudoginseng* is one of the ancestral progenitors of the allopolyploid species (Figure 4A–D).

### 2.4. Whole-Genome Duplication Events in Araliaceae

WGD events can rapidly enlarge the genome within a short time, providing a rich genetic foundation for the adaptive evolution of plants. They are crucial evolutionary events in the process of species evolution. By analyzing the synonymous substitutions (*Ks*) of paralogous genes at each locus in the transcriptome data of Araliaceae plants, we plotted the *Ks* distribution and detected potential WGD events in Araliaceae plants based on the position of the peak. The *Ks* distribution revealed a peak at approximately 0.3 for Aralioideae plants, which may correspond to the pg-β event previously reported in *Panax*. Using the plant-wide synonymous substitution rate of 6.1 × 10^−9^, the peak position corresponds to around 24.6 MYA, which is prior to the divergence of Aralioideae (18 MYA) (Figure 3A). This suggests that Aralioideae plants collectively underwent the pg-β event (Figure 3A,B). However, the peak position of *H. umbellata* differs from that of Aralioideae plants but appears at a similar position to the peak of *C. asiatica* from the Apiaceae (Figure 3B).

**Figure 5:**
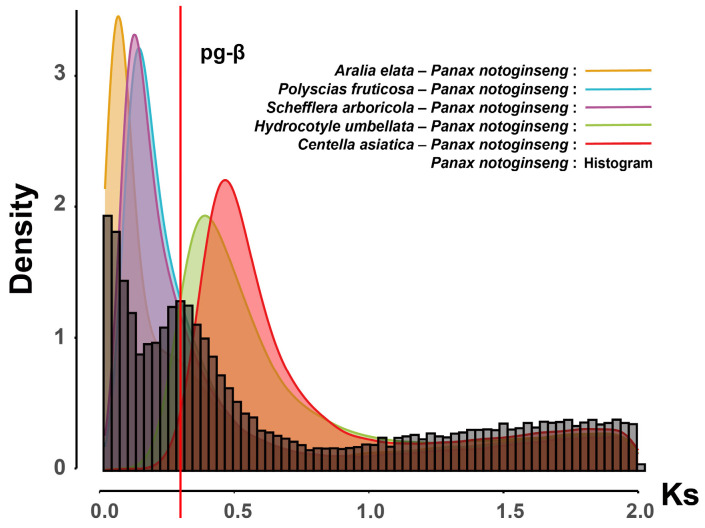
The *Ks* distribution plots between *P. notoginseng* and representative species of each group. The curve plots represent the *Ks* distribution of homologous genes between *P. notoginseng* and representative species of each group. The histogram represents the *Ks* distribution of homologous genes within *P. notoginseng*. The red vertical line indicates the pg-β event.

**Figure 6:**
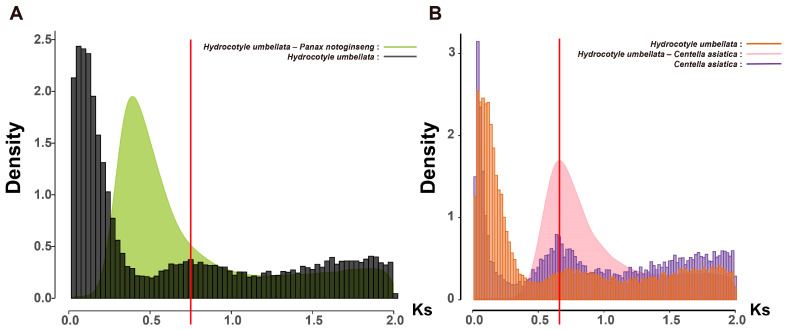
*Ks* distribution of orthologous genes between species. (**A**) *Ks* distribution of orthologous genes between *H. umbellata* and *P. notoginseng*. The line chart represents the *Ks* distribution of orthologous genes between *H. umbellata* and *P. notoginseng*. The black histogram represents the *Ks* distribution of paralogous genes within *H. umbellata*. The red vertical line indicates the position of the WGD event in *H. umbellata*. (**B**) *Ks* distribution of orthologous genes between *H. umbellata* and *C. asiatica* (Apiaceae). The line chart represents the *Ks* distribution of orthologous genes between *H. umbellata* and *C. asiatica*. The orange histogram represents the *Ks* distribution of paralogous genes within *H. umbellata*, while the blue histogram represents the *Ks* distribution of paralogous genes within *C. asiatica*. The red vertical line indicates the peak position of the *Ks* distribution for orthologous genes between *H. umbellata* and *C. asiatica*.

To further investigate whether the pg-β event is a shared WGD event in Araliaceae plants, we used *P. notoginseng* as a reference and calculated the *Ks* values of its orthologous genes with representative plants from various lineages (*Schefflera arboricola*, *Polyscias fruticosa*, *Aralia elata*, *H. umbellata*, and *C. asiatica*) to analyze the relationship between species divergence and the timing of the pg-β event (Figure 5). The results showed that the divergence of Aralioideae plants (*S. arboricola*, *P. fruticosa*, and *A. elata*) from *P. notoginseng* occurred after the pg-β event, while the divergence of *H. umbellata* from *P. notoginseng* took place before the pg-β event. This indicates that Aralioideae plants collectively underwent the pg-β event, while *H. umbellata* did not (Figure 5). The *Ks* peak in *H. umbellata* is positioned prior to its divergence from *P. notoginseng*, but this peak was not observed in other Aralioideae plants (Figure 6A). We speculate that the genes duplicated during this WGD event may have undergone substantial loss throughout the evolutionary history of Aralioideae plants, contributing to their significant morphological differences. Additionally, to determine whether the *Ks* peaks in *C. asiatica* and *H. umbellata* represent the same event, we calculated the *Ks* values of orthologous genes between these two species. The results showed that the *Ks* peak of orthologous genes was similar to the *Ks* peak of paralogous genes within each species (Figure 6B), making it difficult to conclude whether they represent the same event based on this analysis alone. To further clarify whether these peaks correspond to the same event, whole-genome data and chromosomal evolution analyses may be required.

The results of this study confirm that the pg-β event was a shared experience among Aralioideae plants. Previous genomic studies on *P. notoginseng* revealed that multiple enzyme genes involved in ginsenoside biosynthesis were duplicated during the pg-β event, suggesting that this event played a significant role in promoting ginsenoside biosynthesis. Theoretically, all Aralioideae plants should have the ability to synthesize ginsenosides as they collectively experienced the pg-β event. However, ginsenosides are primarily found in large quantities in *Panax* species. Therefore, we conducted an in-depth analysis of the expression patterns of key enzyme genes involved in the ginsenoside biosynthesis pathway in Araliaceae to gain insights into the specific ability of *Panax* species to synthesize ginsenosides within the family.

### 2.5. Gene Expression Patterns in the Ginsenoside Biosynthesis Pathway

**Figure 7:**
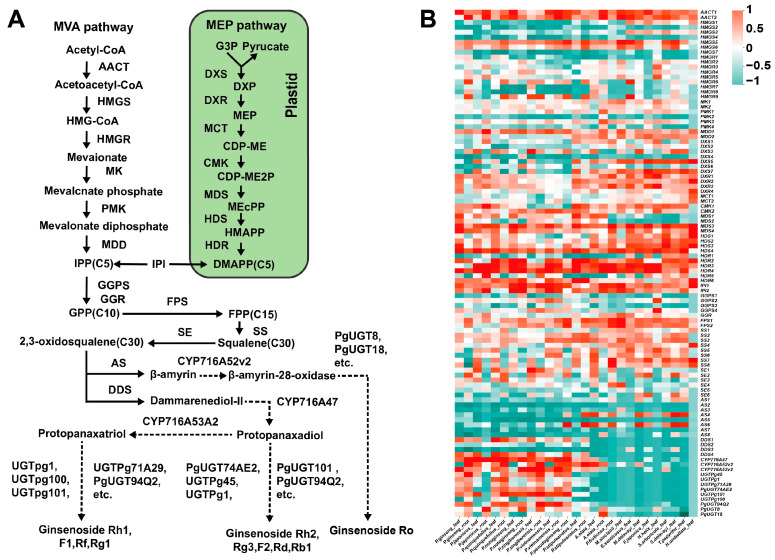
Expression patterns of key genes in the ginsenoside biosynthesis pathway in Araliaceae plants. (**A**) Metabolic pathway of triterpene saponin biosynthesis in *P. ginseng*. (**B**) Expression patterns of 23 key enzyme genes in the ginsenoside biosynthesis pathway. The expression levels are represented by TPM (Transcripts Per Million).

Araliaceae plants produce a wide variety of triterpenoid compounds; however, ginsenosides, triterpenoids with exceptionally high medicinal value, have been detected in significant quantities exclusively in *Panax* species. We explored the expression patterns of key gene families involved in the ginsenoside biosynthesis pathway across different Araliaceae species to identify the enzyme genes responsible for the specific synthesis of ginsenosides in *Panax* species. To achieve this, we used the *Panax* genome as a reference to calculate gene expression levels for various Araliaceae species. Using reference sequences of gene families in the ginsenoside biosynthesis pathway, we identified the members of each gene family within *Panax*. Finally, we compared the expression levels of these gene family members to analyze the expression patterns of key gene families in the ginsenoside biosynthesis pathway across different Araliaceae species. The results revealed that 23 key gene families have multiple copies, with different copies exhibiting distinct expression patterns within the same species. For instance, dammarenediol-II synthase (*DDS*) has three highly expressed copies (*DDS1*, *DDS2*, *DDS4*) in *Panax* species, while one copy (*DDS3*) shows no expression (Figure 7B, Table S7). This complex expression pattern highlights the intricate regulatory mechanisms of key genes in the ginsenoside biosynthesis pathway. The homologous genes of *β-AS* (*β-AS1*, *β-AS4*, and *β-AS6*) are highly expressed in multiple genera but exhibit low expression in most *Panax* species, except in the roots of *Panax stipuleanatus* and *P. pseudoginseng*. In contrast, the three homologous genes of *DDS* (*DDS1*, *DDS2*, and *DDS4*) show the opposite trend (Figure 7B). This divergence in expression patterns may be one of the key reasons why ginsenosides are synthesized exclusively in *Panax* species. Given the large number of members in the cytochrome P450 (*CYP450*) and UDP-glycosyltransferase (*UGTs*) families, this study focused only on genes with confirmed functions in *Panax*. The *CYP450* genes involved in the synthesis of dammarane-type ginsenosides, *CYP716A47* (protopanaxadiol) and *CYP716A53v2* (protopanaxatriol), are highly expressed only in *Panax* species, while the *CYP716A52v2* gene involved in oleanane-type ginsenoside synthesis is also highly expressed in *A. elata* (Figure 7A,B). *UGTs* catalyze the final step of ginsenoside biosynthesis, the glycosylation reaction. Seven *UGTs* genes involved in the synthesis of dammarane-type ginsenosides (*UGTPg45*, *UGTPg1*, *UGTPg71A29*, *PgUGT74AE2*, *UGTPg101*, *UGTPg100*, *PgUGT94Q2*) are highly expressed in *Panax* species but either show no expression or are expressed at low levels in other Araliaceae plants (Figure 7A,B). The *UGT8* and *UGT18* genes involved in the synthesis of oleanane-type ginsenosides are highly expressed not only in *Panax* species but also in the roots of *A. elata* (Figure 7B). The high expression of *CYP716A52v2*, *UGT8*, and *UGT18* genes in *A. elata* suggests that, even with transcriptome data obtained from different studies, their expression patterns can still be accurately reflected to some extent. Based on the differential expression patterns of *β-AS*, *DDS*, *CYP450*, and *UGTs* genes between *Panax* species and other Araliaceae plants, we hypothesize that these genes may be key enzymes responsible for the specific synthesis of ginsenosides in *Panax* species.

**Figure 8:**
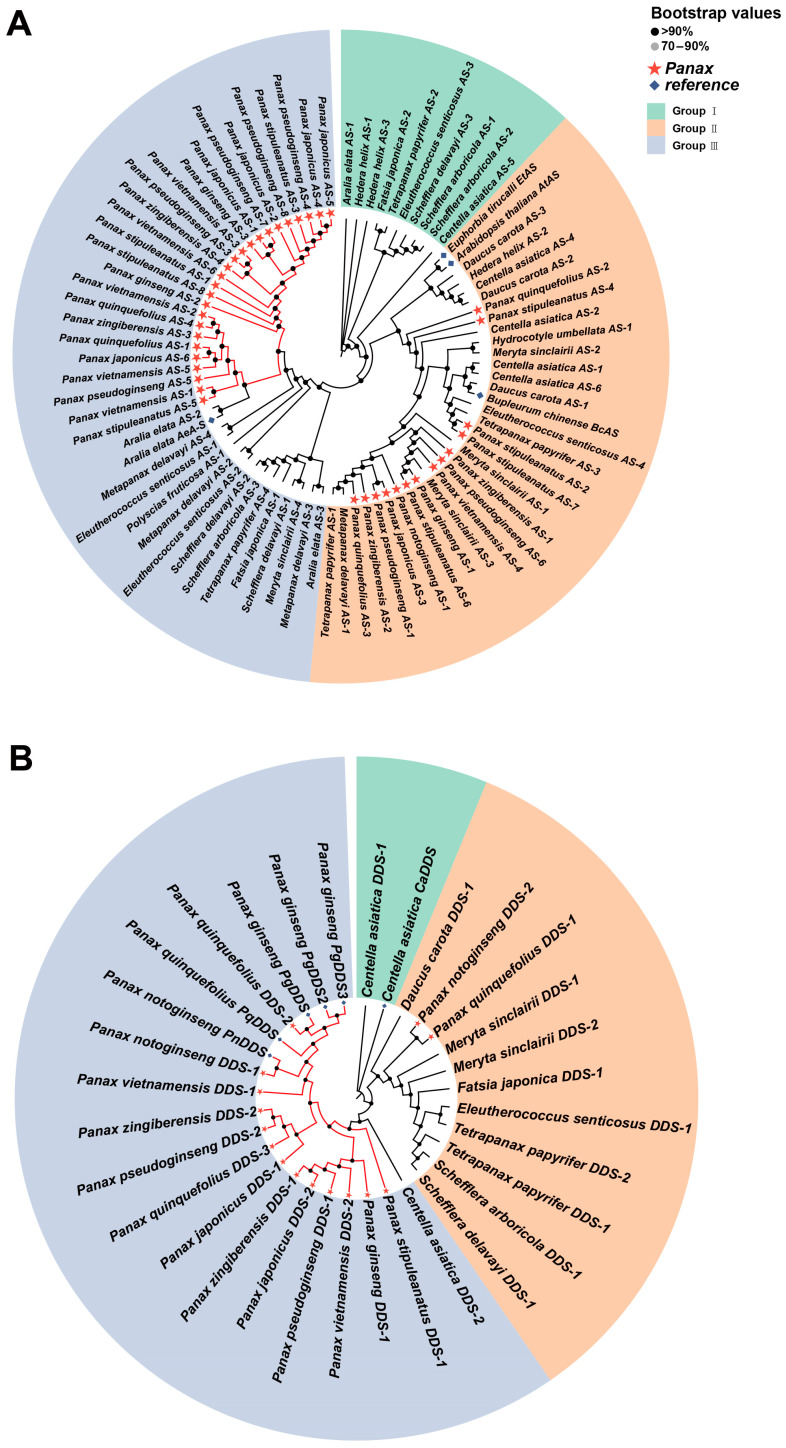
Gene trees of *β-AS* and *DDS* homologous genes in Araliaceae plants. (**A**) Gene tree of *β-AS*. (**B**) Gene tree of *DDS*. The red stars indicate homologous genes from *Panax* plants, and the blue diamonds represent the seed genes used for homologous gene identification of each enzyme gene. The genes are grouped according to the branches of the gene tree. The darker the color of the dot at the node, the higher the support for the node.

**Figure 9:**
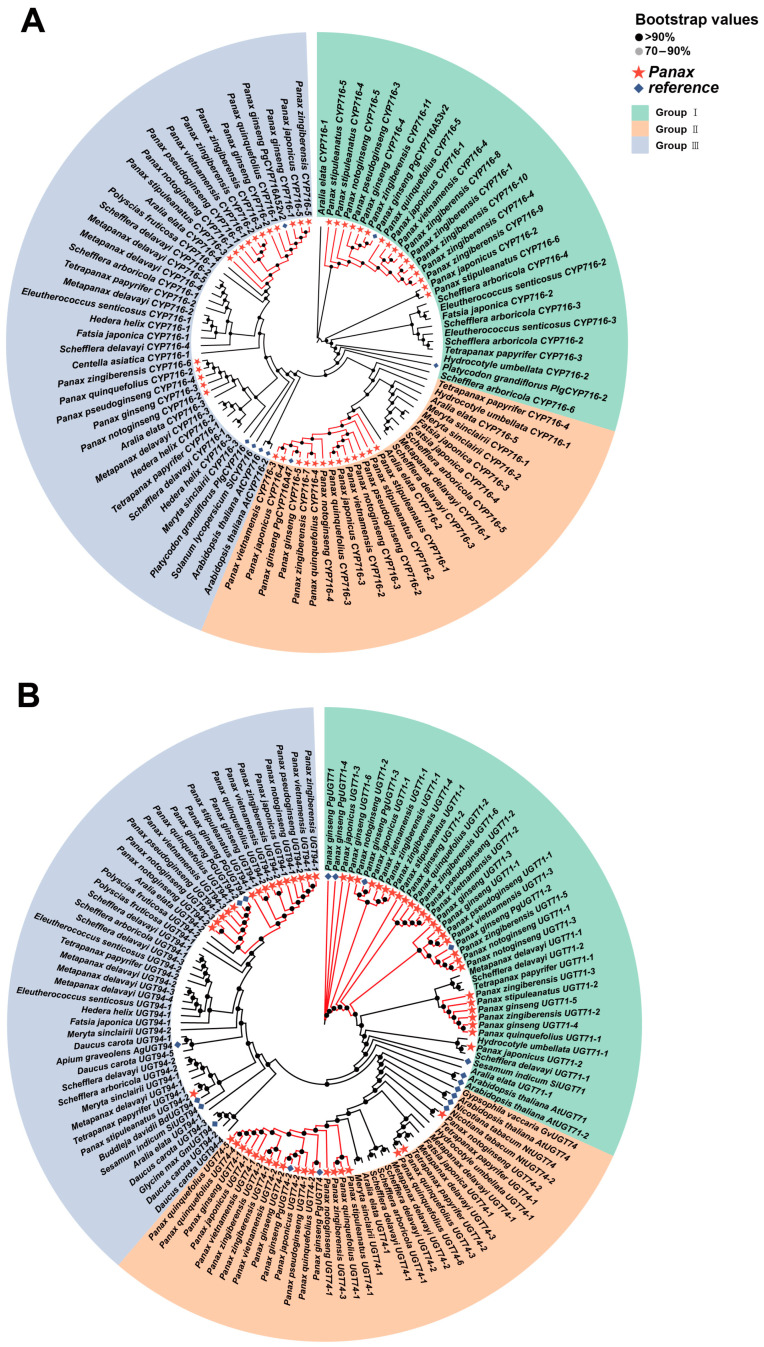
Gene trees of *CYP450* and *UGTs* homologous genes in Araliaceae plants. (**A**) Gene tree of *CYP450*. (**B**) Gene tree of *UGTs*. The red stars indicate homologous genes from *Panax* plants, and the blue diamonds represent the seed genes used for homologous gene identification of each enzyme gene. The genes are grouped according to the branches of the gene tree. The darker the color of the dot at the node, the higher the support for the node.

To investigate the evolutionary relationships of the four key enzyme genes (*β-AS*, *DDS*, *CYP450*, and *UGTs*) in Araliaceae plants, we used genes identified in previous studies as references to identify putative homologous genes in various Araliaceae species and constructed corresponding gene trees. The results showed that Araliaceae plants universally possess homologous genes for these four key enzymes. Notably, the *UGT71* and *UGT74* subfamilies within the *UGTs* family have significantly higher copy numbers in *Panax* species compared to other species (Table S8). In the gene tree constructed for *β-AS* homologous genes, two major clades were identified. One clade contains reference genes from multiple species, while the other clade includes reference genes from *Aralia* clustered with homologous genes identified in *Panax* species. This pattern reflects the close phylogenetic relationship between *Panax* and *Aralia* and suggests that their *β-AS* homologous genes may have similar functions (Figure 8A). In the gene tree constructed for *DDS* homologous genes, homologous genes from *Panax* species formed a distinct clade, with all previously identified *DDS* genes located within this clade. This suggests that *DDS* homologous genes in *Panax* may possess unique functions that differentiate them from those in other species (Figure 8B). As the *CYP716* subfamily plays a major role in ginsenoside biosynthesis, we identified putative homologous genes specifically within this subfamily. In the gene tree of the *CYP716* subfamily, three distinct clades consisting of homologous genes from *Panax* species were observed, with each clade containing a reference gene with a known function in *Panax*. Furthermore, these three clades were nested within different larger branches, suggesting that members of the *CYP716* subfamily with different functions may have distinct evolutionary origins (Figure 9A). In the gene tree of *UGTs*, members of each subfamily clustered together. Most homologous genes of *UGT71* were identified in *Panax* species and formed a distinct cluster, while a clade in *UGT74* consisted entirely of homologous genes from *Panax* species. In *UGT94*, homologous genes from *Panax* were densely distributed within a branch that also included homologous genes from *Aralia*, suggesting that these genes may share similar functions (Figure 9B). In summary, distinct clades consisting of homologous genes from *Panax* species were observed in all four gene trees. This phenomenon suggests that the homologous genes of the four key enzymes may have undergone independent evolutionary processes in *Panax* species, resulting in functions distinct from their homologs in other species. This functional divergence likely enables *Panax* species to synthesize ginsenosides specifically.

## 3. Discussion

### 3.1. Resolution of the Phylogeny of Araliaceae Plants

Previous studies have shown that the morphological classification of Araliaceae plants is quite challenging, and this has been a major barrier to the conservation, development, and sustainable utilization of the germplasm resources of this family, as well as to the related industries [1,12,13]. Although early researchers conducted molecular phylogenetic studies on Araliaceae using the nuclear rRNA gene internal transcribed spacer (ITS) and the trnL-trnF region of the chloroplast genome [1,12,13], the limited data and high sequencing costs have resulted in unclear phylogenetic relationships within the Araliaceae family [1,12,13]. With the rapid development of next-generation sequencing platforms, both genomic and organellar genomes have been applied to the study of phylogenetic relationships in Araliaceae plants. Compared to these methods, transcriptome data provide abundant informative loci and high feasibility, gradually becoming a crucial approach for phylogenetic research. This approach has shown excellent performance in the phylogenetic studies of green plants [17], Leguminosae [19], *Camellia* [16], and other taxa. The use of transcriptome data is limited by RNA degradation and the difficulty in preserving sample data, which restricts its practicality. However, in the study by He et al. (2022) [23] on the phylogeny of Ranunculaceae plants, they successfully sequenced RNA extracted from silica gel-dried plant tissues, successfully resolving the phylogenetic relationships of Ranunculaceae. This result increased the practicality of transcriptome data in resolving phylogenetic relationships. Therefore, the transcriptome data from 19 Araliaceae species and two outgroup species from Apiaceae were used in this study to analyze the phylogenetic relationships of Araliaceae based on these species’ 510 single-copy orthologous genes. Our research reconstructed a robust and well-resolved phylogenetic framework for Araliaceae by two methods, with most branches receiving maximum support [IQ-TREE bootstrap support = 100, MrBayes posterior probability = 1, ASTRAL local posterior probability = 1] (Figure 1). In addition, by comparing with the results obtained by previous researchers using gene fragments and chloroplast genomes, we found that the generic relationships obtained in this study are the same as theirs [2,12,13]. This shows that the phylogenetic relationships constructed using the transcriptome data from 19 species of Araliaceae plants can accurately reflect the true affinity among genera in the family. In summary, transcriptome data has a significant advantage in constructing the phylogenetic relationships of Araliaceae and can provide a clearer and more stable phylogenetic tree.

Our phylogenetic analysis and divergence time study suggest that the Araliaceae family likely originated around 57.29 MYA [95% HPD = 56.5, 58.12], and support the division of Araliaceae into Aralioideae and Hydrocotyloideae around 39.97 MYA [95% HPD = 39.29, 40.64]. Subsequently, the diversification of Aralioideae plants began around 18 MYA [95% HPD = 17.85, 18.16] (Figure 3). Aralioideae is the core group of the Araliaceae family. In our study, we classified Aralioideae into three groups: the *Aralia–Panax* group, the Asian Palmate group, and the *Polyscias–Pseudopanax* group. The *Aralia–Panax* group includes *Panax* and *Aralia*, with *Aralia* positioned as the sister lineage to *Panax* at the base of this clade (Figure 1). The *Polyscias–Pseudopanax* group and the *Aralia–Panax* group were identified as sister clades, consistent with previous studies based on nuclear and plastid genes (Figure 1). For example, Wen et al. (2001) [12], using nuclear ITS sequences, collectively referred to the *Aralia–Panax* group and the *Polyscias–Pseudopanax* group as the *Aralia–Polyscias–Pseudopanax* group. Similarly, Kang et al. (2023) [2], through phylogenetic analysis of plastid genomes, confirmed that the *Polyscias–Pseudopanax* group is the sister clade to the *Aralia–Panax* group. The Asian Palmate group comprises the largest number of species within Araliaceae, including the largest genus, *Schefflera* [2]. Due to the high species diversity and early rapid radiation within the group, its phylogenetic relationships remain unclear. For instance, the phylogenetic positions of *Hedera–Merrilliopanax* and *Kalopanax–Metapanax* clades are yet to be resolved [2,12]. In our study, the limited sample size prevented a comprehensive resolution of the phylogenetic relationships within the Asian Palmate group; however, we successfully established a phylogenetic framework with a stable topology, and the internal branches covering six genera of plants showed very high support (Figure 1). In the future, broader species sampling is expected to further elucidate the complex phylogenetic relationships within this group and provide a more comprehensive understanding of its evolutionary history. Much taxonomic information indicates that the systematic classification of subfamily Hydrocotyloideae has been highly controversial. Hydrocotyloideae was classified under Apiaceae for a long time until Xie et al. (2022) [24] constructed the phylogenetic relationships of Apiales using 72 plastid genes, which confirmed that the genus *Hydrocotyle* belongs to Araliaceae. Our study also further validates this result (Figure 1).

Choi et al. (2014) [25] used fluorescence in situ hybridization (FISH) analysis to demonstrate that *P. ginseng* is an allopolyploid, but lacked evidence of its diploid progenitor. Therefore, we used PhyloNet v3.8.2 [26] analysis to investigate the origin of the parental species of *P. ginseng*. The phylogenetic network of *Panax* that we constructed shows that the common ancestor of *P. ginseng*, *P. japonicus*, and *P. quinquefolius* was an allopolyploid (Figure 4A–D). The phylogenetic network proposed by Zhang et al. (2025) [15] also supports the hybrid origin of a common ancestor for *P. ginseng*, *P. japonicus*, and *P. quinquefolius*. Additionally, Wang et al. (2022) [27] constructed a phylogenetic tree using the subgenomes of *P. ginseng*, *P. japonicus*, and *P. quinquefolius*. The results indicated that the six subgenomes were divided into two branches, with each branch containing subgenomes from all three species. These results indicate that the common ancestor of *P. ginseng*, *P. japonicus*, and *P. quinquefolius* underwent allopolyploidization, becoming an allopolyploid. We hypothesize that the allopolyploid genome of *P. ginseng* originated from their common ancestor, suggesting that *P. japonicus* and *P. quinquefolius* may also be allopolyploids. In the phylogenetic networks of individual allopolyploid *Panax* species and *Panax*, the species involved in hybridization differ, which makes it impossible to directly determine the parent species of the common ancestor of the three allopolyploid species (Figure 4A–D). However, in all four phylogenetic networks, *P. pseudoginseng* is consistently assumed to be one of the progenitors (Figure 4A–D). *P. notoginseng* is assumed to be one of the progenitors in the phylogenetic networks of individual allopolyploid *Panax* species (Figure 4A–C). In the subgenomic phylogenetic analyses by Wang et al. (2022) [27] and Song et al. (2024) [3], it was found that the genome of *P. notoginseng* is closely related to the subgenomes of allopolyploid *Panax* species. Based on the above, we hypothesize that *P. pseudoginseng* and *P. notoginseng* may be the diploid progenitors of the common ancestor of *P. ginseng*, *P. japonicus*, and *P. quinquefolius*. Furthermore, we suggest that the common ancestor of *P. ginseng*, *P. japonicus*, and *P. quinquefolius* may have undergone intermediate hybridization and allopolyploidization in Southeast Asia, where *P. pseudoginseng* and *P. notoginseng* are widely distributed. This hypothesis also supports the “two-step migration hypothesis” which suggests that the ancestor of *P. quinquefolius* underwent tetraploidization in Asia before migrating to North America, where the modern *P. quinquefolius* evolved.

Previous studies have analyzed WGD events in a few Araliaceae plants, such as *P. ginseng*, *P. notoginseng*, *P. quinquefolius* from *Panax*, and *Eleutherococcu senticosus* and *A. elata* from the *Eleutherococcus* and *Aralia* genera [3,27,28,29,30]. However, it remains unclear whether these WGD events are shared across all Araliaceae plants or are specific to certain species. Therefore, we analyzed WGD events in the aforementioned 21 species by calculating the *Ks* values of paralogous genes within species. Our results indicate that Aralioideae plants universally experienced the pg-β event (Figure 3 and Figure 5), a finding also reported in genomic studies of *P. ginseng*, *P. notoginseng, E. senticosus*, and *A. elata* [3,28,29,30]. This study, using data from a broader range of species, provides further evidence supporting this hypothesis. In addition to the pg-β event, some Araliaceae plants have experienced other WGD events; for example, the pg-α event was detected in *P. ginseng*, and the Es-α event was identified in *E. senticosus* species [3,28]. Following these recent WGD events, both species were found to be allopolyploids, and no new WGD events have been identified in other Araliaceae species thus far [3,14,28,31]. Therefore, we hypothesize that post-WGD rediploidization and hybridization events may have been the primary drivers of diversification in Aralioideae plants.

In our study, the *Ks* peak of *H. umbellata* differs from that of Aralioideae plants, and this peak occurred prior to the divergence between *H. umbellata* and *P. notoginseng* (Figure 3 and Figure 6A). Therefore, we hypothesize that this WGD event might have been experienced by all Araliaceae plants, but during the evolution of Aralioideae, a substantial loss of genes resulting from this event may have occurred, leading to significant phenotypic differences between Hydrocotyloideae and Aralioideae plants. That might explain why early morphological classifications consistently placed Hydrocotyloideae within Apiaceae. In our study, only *H. umbellata*, a species from the Hydrocotyloideae subfamily, was included, and no closely related species were available as a reference. Therefore, we cannot confirm this as a newly discovered WGD event. However, this is an intriguing discovery that warrants further investigation. Future studies with additional Hydrocotyloideae species are needed to verify our hypothesis.

### 3.2. The Expression Patterns and Evolutionary Relationships of Key Enzyme-Coding Genes in Ginsenoside Biosynthesis of Araliaceae Plants

Araliaceae plants can synthesize a wide variety of triterpenoid compounds, but only *Panax* species can synthesize ginsenosides in large quantities [32]. Understanding the reasons behind this phenomenon is crucial for the development and utilization of Araliaceae plants. To this end, we utilized transcriptome data from 19 Araliaceae species to construct the expression patterns of key enzyme genes involved in the ginsenoside biosynthesis pathway in Araliaceae plants. By analyzing the expression patterns of key enzyme-coding genes involved in the ginsenoside biosynthesis pathway in Araliaceae, we found that the expression patterns of *AS*, *DDS*, *CYP450*, and *UGTs* genes in *Panax* species differ from those in other Araliaceae plants (Figure 7B). *DDS* and *AS*, both belonging to the oxidosqualene cyclase (*OSC*) family, are involved in the cyclization of 2,3-oxidosqualene, forming the basic skeleton of ginsenosides [33]. DDS has been confirmed as a key enzyme in the synthesis of dammarane-type ginsenosides, with its overexpression or introduction shown to increase ginsenoside production [34]. AS is the key enzyme for synthesizing oleanane-type ginsenosides [35]. Additionally, silenced expression of *DDS* leads to increased expression of other *OSC* members, explaining the phenomenon of low *AS* expression in plants with high *DDS* expression. The phylogenetic analysis of genes shows that homologous *AS* genes in *Aralia* cluster with those in *Panax* species, while *DDS* homologs in *Panax* form a distinct clade, including reference genes with verified functions (Figure 8A,B). This indicates that the *DDS* gene in *Panax* species may have undergone independent evolution, resulting in the gene’s neofunctionalization, which ultimately led to the large-scale synthesis and accumulation of ginsenosides in *Panax* species [36]. These results further confirm the findings in our previous research. Studies have shown that in *Aralia*, the loss of exons in *DDS*-encoding genes leads to the inability to produce dammarane-type saponins, while the introduction of the *PgDDS* gene from *P. ginseng* can restore the accumulation of dammarane-type saponins in *Aralia* callus tissues [37]. Additionally, Yang et al. (2023) [38], through genomic and biochemical analyses, demonstrated that *OSC* genes responsible for dammarane-type saponin synthesis were generated during the pg-β event. Therefore, we hypothesize that after the pg-β event, other Aralioideae plants outside *Panax* may have experienced *DDS* gene loss or gene fragment deletions during their evolutionary process, leading to low or absent expression of the *DDS* gene. This, in turn, would prevent the large-scale synthesis of dammarane-type triterpenoid skeletons and affect the synthesis of ginsenosides.

CYP450 primarily participates in oxidation and hydroxylation reactions during ginsenoside biosynthesis [39], while UGTs function in the final step of ginsenoside biosynthesis. UGTs transfer glycosyl residues from activated sugars to the aglycones of ginsenosides, thereby regulating the biological activity, solubility, and stability of ginsenosides. Previous studies have shown that the *CYP450* and *UGTs* gene superfamilies are widespread in plants and have a large number of members; for example, *P. ginseng* contains 550 *CYP450* genes and 273 *UGTs* genes [3]. In *P. ginseng*, three members of the *CYP450* superfamily have been identified as being involved in the regulation of ginsenoside biosynthesis. Specifically, *CYP716A47* regulates the synthesis of protopanaxadiol-type ginsenosides, *CYP716A53v2* regulates the synthesis of protopanaxatriol-type ginsenosides, and *CYP716A52v2* regulates the synthesis of oleanane-type ginsenosides [7,32,40]. In addition to being highly expressed in *Panax* species, *CYP716A52v2* also exhibits significantly high expression in *Aralia*. *CYP716A52v2* is a β-amyrin-28-oxidase that converts β-amyrin into oleanolic acid, which serves as the aglycone of ginsenoside Ro [41]. The *UGT8* and *UGT18* genes, which are involved in the synthesis of ginsenoside Ro, also exhibit high expression in the roots of *A. elata* (Figure 7B). This is consistent with the presence of ginsenoside Ro in *A. elata* [42]. Furthermore, the *CYP450* gene tree shows that the homologous genes of *A. elata* cluster within the same clade as those of *Panax* species (Figure 9A). These results suggest that the homologous genes in *A. elata*, which are in the same clade as those in *Panax* species, may share similar functions. *UGTs* are widely involved in the glycosylation of terpenoids [43]. In our study, *UGTs* participating in ginsenoside biosynthesis were either not expressed or expressed at extremely low levels in species outside *Panax*. The number of homologous *UGTs* genes identified in the transcriptomes of *Panax* species was significantly higher than in other species, with the *UGT71* and *UGT74* subfamilies showing the most pronounced differences (Table S8). The gene tree further revealed that putative homologous genes in *Panax* clustered together, indicating that these genes have undergone independent evolution within *Panax* (Figure 9B). Yang et al. (2021) [28] reported that *E. senticosus* contains 125 *UGTs* members, notably fewer than the 144 found in *P. notoginseng*. Therefore, we speculate that additional gene duplication events occurred during the evolution of *Panax*, leading to an increased number of functional *UGTs*. Moreover, some *UGTs* genes have also undergone independent evolution within *Panax*. These evolutionary events not only led to the large-scale synthesis of ginsenosides in *Panax* species but also increased the diversity of ginsenosides. These uniquely evolved and specifically expressed genes should be considered important candidate genes for subsequent functional validation experiments.

## 4. Materials and Methods

### 4.1. Data Sources and Transcriptome Assembly

In this study, we collected transcriptome data of all extant Araliaceae plants from public databases and successfully obtained data from 19 species (11 genera). We used *C. asiatica* and *D. carota* from Apiaceae as outgroups, giving a total of 21 species’ data. All data are from GenBank SRA (Table S9). We deeply explored the phylogenetic relationships of the 19 Araliaceae species based on these data. Quality assessment of the raw transcriptomic reads was performed using FastQC v0.12.1 [44]. Subsequently, low-quality bases, adapter sequences, and potential contaminants were removed from the raw reads using Trimmomatic v0.39 [45], resulting in clean reads.

Clean reads were assembled de novo into transcriptomes using Trinity v2.15.1 [46] with default parameters. The resulting transcripts were processed with CD-HIT v4.8.1 [47] to remove highly similar sequences, applying the parameters “-c 0.98” and “-n 5”. The completeness of the assembly was evaluated using BUSCO v5.4.7 [48] with the embryophyta_odb10 database. Candidate coding regions were identified from the assembled transcripts using TransDecoder v5.7.1 [46].

Initially, the “TransDecoder.LongOrfs” command was used to predict open reading frames (ORFs), selecting those with a length of at least 100 amino acids. Subsequently, the amino acid sequences of these ORFs were aligned against protein databases constructed from *P. ginseng* [3] and *Arabidopsis thaliana* [49] using BLASTP v2.5.0 [50], with parameters set to “-evalue 1 × 10^−5^” and “-max_target_seqs 1” to identify the best matches. Finally, the “TransDecoder.Predict” command was used to integrate the BLASTP v2.5.0 [50] search results in order to predict the most likely expressed nucleotide coding sequences and their corresponding protein sequences.

### 4.2. Gene Functional Annotation and Single-Copy Orthologue Identification

Functional annotation of Araliaceae transcripts was performed by aligning the predicted protein sequences against seven widely recognized protein databases, including Swiss-Prot, PFAM, GO, COG, KEGG, NR and TAIR11. The alignment was conducted using BLASTP v2.5.0 [50] with a threshold parameter of “-evalue 1 × 10^−5^”. TFs in Araliaceae were predicted and classified using the ITAK v1.6 tool [51] with default parameters.

Single-copy orthologous genes were identified from the protein dataset predicted by TransDecoder v5.7.1 [46]. To minimize redundancy, the longest sequences showing homology to protein sequences derived from the *P. ginseng* and *A. thaliana* genomes were selected. Subsequently, these filtered protein sequences were processed through the OrthoFinder v2.5.5 pipeline [52] with default parameters to cluster orthologous groups. The final phylogenetic analysis was based on the “Single_Copy_Orthologue_Sequences” subset from OrthoFinder v2.5.5 [52] results, which strictly retained only orthogroups containing all target species with exactly one gene copy per species to ensure phylogenetic reliability [16].

### 4.3. Phylogeny Construction and Divergence Time Estimation

This study reconstructs the phylogenetic relationships of Araliaceae plants using both concatenation-based and coalescent-based approaches [16,17,18]. For the concatenation method, MUSCLE v5.1 [53] with default parameters was employed to align each single-copy orthologous gene, and poorly aligned regions were trimmed using trimAl v1.4.rev15 [54]. The alignment results were then concatenated into a supergene sequence matrix using Python v3.12.0 scripts. Subsequently, phylogenetic trees were constructed using the maximum likelihood method in IQ-TREE v2.2.5 [55] and the Bayesian inference method in MrBayes v3.2.7a [56]. When constructing the phylogenetic tree with IQ-TREE v2.2.5 [55], the parameter “-m MFP+MERGE” was used to automatically detect the best substitution model for each partition, and support values were calculated using 1000 bootstrap replicates. In constructing the phylogenetic tree using MrBayes v3.2.7a [56], the best substitution model for each partition was determined using IQ-TREE v2.2.5 [55] with the “-m TESTMERGEONLY” option. The “-mset mrbayes” option was applied to ensure that the selected models were compatible with MrBayes v3.2.7a [56]. During the execution of MrBayes v3.2.7a [56], three independent MCMC chains were set up with a total of 100,000 iterations, and sampling was performed every 1000 iterations. For the coalescent-based method, individual gene trees were constructed using IQ-TREE v2.2.5 [55], and species trees were inferred by integrating the gene trees using ASTRAL v5.7.8 [57]. Gene tree conflicts along the topology of the species tree were assessed using PhyParts v0.0.1 [58] with default parameters. Visualization of these conflicts was performed using the phypartspiecharts.py script provided by the program’s authors.

We used the supergene sequence obtained from the concatenation method as input for the MCMCTree module in PAML v4.10.7 [59] to estimate the divergence times of Araliaceae species. Based on prior research, we selected three nodes for calibration. First, per Yang et al. (2021) [28], we set the diversification start time of the subfamily Aralioidae at 18–19 MYA, based on the divergence time between *P. ginseng* and *E. senticosus*. Second, following Choi et al. (2013) [60], we set the divergence time between *P. ginseng* and *P. quinquefolius* at 0.8–1.2 MYA. Third, per Kang et al. (2023) [2], we constrained the divergence time between the Apiaceae and Araliaceae families to within 69 MYA. Finally, the species tree and divergence time tree were visualized using Chiplot [61].

### 4.4. Inferring the Origin of Allopolyploidy in the Panax Genus

This study utilized the “InferNetwork_MP_Allopp” command in PhyloNet v3.8.2 [26] to infer the putative ancestors of tetraploids within the *Panax* species [22]. This method applies a parsimony-based approach under the Minimizing Deep Coalescences (MDC) criterion to infer allopolyploid species networks. From the 510 single-copy orthologous gene sequences identified in Araliaceae, sequences from eight *Panax* species and *Aralia* were extracted. When constructing gene trees, *Aralia* was used as the outgroup. When inferring the putative ancestors of tetraploids within *Panax*, phylogenetic networks were constructed using *P. ginseng*, *P. quinquefolius*, and *P. japonicus* alongside other diploid *Panax* species. The corresponding tetraploid species were designated as potential hybrid taxa, with a single hybridization event allowed. We also constructed a phylogenetic network for *Panax* species, designating *P. ginseng*, *P. quinquefolius*, and *P. japonicus* as potential hybrid taxa and allowing for up to three hybridization events. Additionally, a phylogenetic network comprising only diploid *Panax* species was constructed using the “InferNetwork_MPL” method in PhyloNet v3.8.2 [26], without specifying potential hybrid taxa and allowing for up to five hybridization events. The resulting phylogenetic networks were visualized using Dendroscope v3.8.10 [62].

### 4.5. Identification of Whole-Genome Duplication Events in Araliaceae

This study detected potential WGD events in Araliaceae plants by analyzing the distribution of *Ks* at each locus of paralogous genes in the transcriptome data [63]. First, redundant protein sequences predicted by TransDecoder v5.7.1 [46] were processed using CD-HIT v4.8.1 [47]. Next, the Python v3.12.0 script ks_plot.py was used to invoke the codeml program from PAML v4.10.7 [59], applying the NG correction method to calculate the *Ks* values for paralogous gene pairs. Finally, the *Ks* value distribution curve was plotted using the ggplot2 package in R v4.2.2 to visually depict potential WGD events. To estimate the timing of WGD events, we inferred the age of the duplication events using the formula Tdiversity = *Ks*/2r, with r = 6.1 × 10^−9^ [36].

### 4.6. Analysis of Expression Patterns of Key Genes in the Ginsenoside Biosynthesis Pathway

To assess the gene expression levels of Araliaceae species, this study used the *P. ginseng* genome as a reference to calculate gene expression levels [64]. First, an index was constructed for the *P. ginseng* genome using HISAT2 v2.2.1 [65], and the Araliaceae transcriptome data were aligned to this genome. The resulting SAM files were converted to BAM files using Samtools v1.15.1 [66], followed by gene-level quantification using featureCounts v2.16.1 [67], with gene expression levels assessed using TPM (transcripts per million). To analyze the expression of key genes in the ginsenoside biosynthesis pathway, reference sequences of these key genes were downloaded from the National Center for Biotechnology Information (NCBI), Pfam, and UniProt databases (Table S10). BLAST v2.5.0 [50] and HMMER v3.4 [68] were then used to align the nucleotide and protein sequences of these reference genes, respectively, in order to select the best matches based on coverage, similarity, and alignment scores. Expression data (TPM) for these genes in Araliaceae species were extracted and normalized using log2(TPM+1). Finally, a heatmap of the key gene expression patterns was generated using Chiplot [61] to visually display the expression profiles of key genes in the ginsenoside biosynthesis pathway.

Based on the expression patterns of key genes, we further investigated the evolutionary trajectories of *DDS*, *β-AS*, *CYP450*, and *UGTs* in Araliaceae species. Functional coding sequences were retrieved from the NCBI as references [69]. Due to the large number of members in the *CYP450* and *UGTs* superfamilies, we focused only on the families primarily involved in ginsenoside biosynthesis. In *P. ginseng*, the *CYP450* genes identified as participating in ginsenoside biosynthesis are concentrated in the *CYP71* family, while most *UGTs* genes involved in ginsenoside biosynthesis belong to the *UGT74*, *UGT94*, and *UGT71* families [6]. Homologous genes from the Araliaceae transcriptomes were identified using BLAST v2.5.0 [50] with the threshold parameter set to “-evalue 1 × 10^−10^”. The BLAST-identified homologous genes were further screened by examining their conserved domains using CD-Search [70]. Sequence alignment for gene tree construction was performed using MUSCLE v5.1 [53], followed by phylogenetic tree construction with IQ-TREE v2.2.5 [55]. The model parameter was set to “-m MFP” for automatic detection of the best substitution model, and bootstrap support values were calculated with 1000 replicates. The resulting gene trees were visualized using Chiplot [61].

## 5. Conclusions

In conclusion, by utilizing transcriptome data from Araliaceae plants and combining comparative transcriptomics with phylogenomic approaches, we provide novel insights into an in-depth analysis of the phylogenetic relationships and evolutionary history of Araliaceae. Furthermore, we investigated the expression patterns and evolutionary relationship of key genes involved in the ginsenoside biosynthesis pathway, providing valuable insights into the specific synthesis of ginsenosides in *Panax* species within the Araliaceae family. This study not only enhances our understanding of the phylogenetic relationships and evolutionary history of Araliaceae, providing a solid scientific basis for the conservation of endangered species and molecular breeding within the family, but also offers important theoretical foundations for research on the biosynthesis and metabolic engineering of ginsenosides.

## Data Availability

The data presented in this study are available in this article and the Supplementary Materials.

## Supplementary Materials

The following supporting information can be downloaded at: https://www.mdpi.com/article/10.3390/ijms26073439/s1.

## References

[1] Li R., Wen J. (2016). Phylogeny and diversification of Chinese Araliaceae based on nuclear and plastid DNA sequence data. J. Syst. Evol..

[2] Kang J.S., Giang V.N.L., Park H.S., Park Y.S., Cho W.H.Y., Nguyen V., Shim H., Waminal N.E., Park J.Y., Kim H.H. (2023). Evolution of the Araliaceae family involved rapid diversification of the Asian Palmate group and *Hydrocotyle* specific mutational pressure. Sci. Rep..

[3] Song Y.T., Zhang Y.T., Wang X., Yu X.K., Liao Y., Zhang H., Li L.F., Wang Y.P., Liu B., Li W. (2024). Telomere-to-telomere reference genome for *Panax ginseng* highlights the evolution of saponin biosynthesis. Hortic. Res..

[4] Wang T., Guo R.X., Zhou G.H., Zhou X.D., Kou Z.Z., Sui F., Li C., Tang L.Y., Wang Z.J. (2016). Traditional uses, botany, phytochemistry, pharmacology and toxicology of *Panax notoginseng* (Burk.) FH Chen: A review. J. Ethnopharmacol..

[5] Yang L., Hou A.J., Zhang J.X., Wang S., Man W.J., Yu H., Zheng S.W., Wang X.J., Liu S.T., Jiang H. (2020). Panacis Quinquefolii Radix: A Review of the Botany, Phytochemistry, Quality Control, Pharmacology, Toxicology and Industrial Applications Research Progress. Front. Pharmacol..

[6] Hou M.Q., Wang R.F., Zhao S.J., Wang Z.T. (2021). Ginsenosides in *Panax* genus and their biosynthesis. Acta Pharm. Sin. B.

[7] Yang J.L., Hu Z.F., Zhang T.T., Gu A.D., Gong T., Zhu P. (2018). Progress on the Studies of the Key Enzymes of Ginsenoside Biosynthesis. Molecules.

[8] Pena R.D., Hodgson H., Liu J.C.T., Stephenson M.J., Martin A.C., Owen C., Harkess A., Leebens-Mack J., Jimenez L.E., Osbourn A. (2023). Complex scaffold remodeling in plant triterpene biosynthesis. Science.

[9] Jiang Z., Ma K. (2017). The state’s will, scientific decision and citizen participation: In memory of the first provincial species red list in China. Biodivers. Sci..

[10] Fang X.X., Wang M.Q., Zhou X.T., Wang H., Wang H.Y., Xiao H.X. (2022). Effects of growth years on ginsenoside biosynthesis of wild *ginseng* and cultivated ginseng. BMC Genom..

[11] Harms H., Engler A., Prantl K. (1898). Araliaceae. Die Natürlichen Pflanzenfamilien.

[12] Wen J., Plunkett G.M., Mitchell A.D., Wagstaff S.J. (2001). The Evolution of Araliaceae: A Phylogenetic Analysis Based on ITS Sequences of Nuclear Ribosomal DNA. Syst. Bot..

[13] Plunkett G.M., Wen J., Lowry P.P. (2004). Infrafamilial classifications and characters in Araliaceae: Insights from the phylogenetic analysis of nuclear (ITS) and plastid (trnL-trnF) sequence data. Plant Syst. Evol..

[14] Liu J., Nie Z.L., Ren C., Su C., Wen J. (2023). Phylogenomics of *Aralia* sect. Aralia (Araliaceae): Signals of hybridization and insights into its species delimitations and intercontinental biogeography. Mol. Phylogenet. Evol..

[15] Zhang M.H., Nie Z.L., Fairbanks R.A., Liu J., Literman R., Johnson G., Handy S., Wen J. (2025). Phylogenomic insights into species relationships, reticulate evolution, and biogeographic diversification of the ginseng genus *Panax* (Araliaceae), with an emphasis on the diversification in the Himalayan-Hengduan Mountains. J. Syst. Evol..

[16] Wu Q., Tong W., Zhao H.J., Ge R.H., Li R.P., Huang J., Li F.D., Wang Y.L., Mallano A.I., Deng W.W. (2022). Comparative transcriptomic analysis unveils the deep phylogeny and secondary metabolite evolution of 116 *Camellia* plants. Plant J..

[17] Leebens-Mack J.H., Barker M.S., Carpenter E.J., Deyholos M.K., Gitzendanner M.A., Graham S.W., Grosse I., Li Z., Melkonian M., Mirarab S. (2019). One thousand plant transcriptomes and the phylogenomics of green plants. Nature.

[18] Meng G.L., Zhou C.R., Liu S.L., Zhou X. (2021). Phylogenomics based on transcriptomics and short-read sequences. Bio-101.

[19] Zhao Y.Y., Zhang R., Jiang K.W., Qi J., Hu Y., Guo J., Zhu R.B., Zhang T.K., Egan A.N., Yi T.S. (2021). Nuclear phylotranscriptomics and phylogenomics support numerous polyploidization events and hypotheses for the evolution of rhizobial nitrogen-fixing symbiosis in Fabaceae. Mol. Plant.

[20] Zhang C.F., Huang C.H., Liu M., Hu Y., Panero J.L., Luebert F., Gao T.G., Ma H. (2021). Phylotranscriptomic insights into Asteraceae diversity, polyploidy, and morphological innovation. J. Integr. Plant Biol..

[21] Won S.Y., Kwon S.J., Lee T.H., Jung J.A., Kim J.S., Kang S.H., Sohn S.H. (2017). Comparative transcriptome analysis reveals whole-genome duplications and gene selection patterns in cultivated and wild *Chrysanthemum* species. Plant Mol. Biol..

[22] Wang S.B., Gao J.P., Li Z.W., Chen K., Pu W.X., Feng C. (2023). Phylotranscriptomics supports numerous polyploidization events and phylogenetic relationships in *Nicotiana*. Front. Plant Sci..

[23] He J., Lyu R., Luo Y.K., Xiao J.M., Xie L., Wen J., Li W.H., Pei L.Y., Cheng J. (2022). A phylotranscriptome study using silica gel-dried leaf tissues produces an updated robust phylogeny of Ranunculaceae. Mol. Phylogenet. Evol..

[24] Xie D.F., Xie C.A., Ren T., Song B.N., Zhou S.D., He X.J. (2022). Plastid phylogenomic insights into relationships, divergence, and evolution of Apiales. Planta.

[25] Choi H.I., Waminal N.E., Park H.M., Kim N.H., Choi B.S., Park M., Choi D., Lim Y.P., Kwon S.J., Park B.S. (2014). Major repeat components covering one-third of the ginseng (*Panax ginseng* C.A. Meyer) genome and evidence for allotetraploidy. Plant J..

[26] Wen D.Q., Yu Y., Zhu J.F., Nakhleh L. (2018). Inferring Phylogenetic Networks Using PhyloNet. Syst. Biol..

[27] Wang Z.H., Wang X.F., Lu T.Y., Li M.R., Jiang P., Zhao J., Liu S.T., Fu X.Q., Wendel J.F., Van de Peer Y. (2022). Reshuffling of the ancestral core-eudicot genome shaped chromatin topology and epigenetic modification in *Panax*. Nat. Commun..

[28] Yang Z.J., Chen S.S., Wang S.F., Hu Y., Zhang G.H., Dong Y., Yang S.C., Miao J.H., Chen W., Sheng J. (2021). Chromosomal-scale genome assembly of *Eleutherococcus senticosus* provides insights into chromosome evolution in Araliaceae. Mol. Ecol. Resour..

[29] Zhang D., Li W., Xia E.H., Zhang Q.J., Liu Y., Zhang Y., Tong Y., Zhao Y., Niu Y.C., Xu J.H. (2017). The Medicinal Herb *Panax notoginseng* Genome Provides Insights into Ginsenoside Biosynthesis and Genome Evolution. Mol. Plant.

[30] Liu W.X., Guo W.H., Chen S., Xu H.H., Zhao Y., Chen S., You X.L. (2022). A High-Quality Reference Genome Sequence and Genetic Transformation System of *Aralia elata*. Front. Plant Sci..

[31] Gallego-Narbón A., Wen J., Liu J., Valcárcel V. (2022). Hybridization and genome duplication for early evolutionary success in the Asian Palmate group of Araliaceae. J. Syst. Evol..

[32] Wang Y., Zhang H., Ri H.C., An Z.Y., Wang X., Zhou J.N., Zheng D.R., Wu H., Wang P.C., Yang J.F. (2022). Deletion and tandem duplications of biosynthetic genes drive the diversity of triterpenoids in *Aralia elata*. Nat. Commun..

[33] Han J.Y., Kwon Y.S., Yang D.C., Jung Y.R., Choi Y.E. (2006). Expression and RNA interference-induced silencing of the dammarenediol synthase gene in Panax ginseng. Plant Cell Physiol..

[34] Han J.Y., Kim H.J., Kwon Y.S., Choi Y.E. (2011). The Cyt P450 Enzyme CYP716A47 Catalyzes the Formation of Protopanaxadiol from Dammarenediol-II During Ginsenoside Biosynthesis in *Panax ginseng*. Plant Cell Physiol..

[35] Kushiro T., Shibuya M., Ebizuka Y. (1998). Beta-amyrin synthase—cloning of oxidosqualene cyclase that catalyzes the formation of the most popular triterpene among higher plants. Eur. J. Biochem..

[36] Zhang D., Li W., Chen Z.J., Wei F.G., Liu Y.L., Gao L.Z. (2020). SMRT- and Illumina-based RNA-seq analyses unveil the ginsinoside biosynthesis and transcriptomic complexity in *Panax notoginseng*. Sci. Rep..

[37] Mohanan P., Yang T.-J., Song Y.H. (2023). Genes and Regulatory Mechanisms for Ginsenoside Biosynthesis. J. Plant Biol..

[38] Yang Z.J., Li X.B., Yang L., Peng S.F., Song W.L., Lin Y., Xiang G.S., Li Y., Ye S., Ma C.H. (2023). Comparative genomics reveals the diversification of triterpenoid biosynthesis and origin of ocotillol- type triterpenes in *Panax*. Plant Commun..

[39] Han J.Y., Hwang H.S., Choi S.W., Kim H.J., Choi Y.E. (2012). Cytochrome P450 *CYP716A53v2* Catalyzes the Formation of Protopanaxatriol from Protopanaxadiol During Ginsenoside Biosynthesis in *Panax ginseng*. Plant Cell Physiol..

[40] Yuan X.X., Li R.Q., He W.S., Xu W., Xu W., Yan G.H., Xu S.H., Chen L.X., Feng Y.Q., Li H. (2024). Progress in Identification of UDP-Glycosyltransferases for Ginsenoside Biosynthesis. J. Nat. Prod..

[41] Han J.Y., Kim M.J., Ban Y.W., Hwang H.S., Choi Y.E. (2013). The Involvement of β-Amyrin 28-Oxidase (*CYP716A52v2*) in Oleanane-Type Ginsenoside Biosynthesis in *Panax ginseng*. Plant Cell Physiol..

[42] Xu Y., Liu J.Y., Zeng Y.L., Jin S.R., Liu W.T., Li Z.L., Qin X.H., Bai Y.L. (2022). Traditional uses, phytochemistry, pharmacology, toxicity and quality control of medicinal genus *Aralia*: A review. J. Ethnopharmacol..

[43] Lu X., Huang L.J., Scheller H., Keasling J.D. (2023). Medicinal terpenoid UDP-glycosyltransferases in plants: Recent advances and research strategies. J. Exp. Bot..

[44] de Sena Brandine G., Smith A.D. (2019). Falco: High-speed FastQC emulation for quality control of sequencing data. F1000Research.

[45] Bolger A.M., Lohse M., Usadel B. (2014). Trimmomatic: A flexible trimmer for Illumina sequence data. Bioinformatics.

[46] Haas B.J., Papanicolaou A., Yassour M., Grabherr M., Blood P.D., Bowden J., Couger M.B., Eccles D., Li B., Lieber M. (2013). De novo transcript sequence reconstruction from RNA-seq using the Trinity platform for reference generation and analysis. Nat. Protoc..

[47] Fu L.M., Niu B.F., Zhu Z.W., Wu S.T., Li W.Z. (2012). CD-HIT: Accelerated for clustering the next-generation sequencing data. Bioinformatics.

[48] Simao F.A., Waterhouse R.M., Ioannidis P., Kriventseva E.V., Zdobnov E.M. (2015). BUSCO: Assessing genome assembly and annotation completeness with single-copy orthologs. Bioinformatics.

[49] Cheng C.Y., Krishnakumar V., Chan A.P., Thibaud-Nissen F., Schobel S., Town C.D. (2017). Araport11: A complete reannotation of the *Arabidopsis thaliana* reference genome. Plant J..

[50] Altschul S.F., Madden T.L., Schaffer A.A., Zhang J., Zhang Z., Miller W., Lipman D.J. (1997). Gapped BLAST and PSI-BLAST: A new generation of protein database search programs. Nucleic Acids Res..

[51] Zheng Y., Jiao C., Sun H.H., Rosli H.G., Pombo M.A., Zhang P.F., Banf M., Dai X.B., Martin G.B., Giovannoni J.J. (2016). iTAK: A Program for Genome-wide Prediction and Classification of Plant Transcription Factors, Transcriptional Regulators, and Protein Kinases. Mol. Plant..

[52] Emms D.M., Kelly S. (2019). OrthoFinder: Phylogenetic orthology inference for comparative genomics. Genome Biol..

[53] Edgar R.C. (2004). MUSCLE: Multiple sequence alignment with high accuracy and high throughput. Nucleic Acids Res..

[54] Capella-Gutiérrez S., Silla-Martínez J.M., Gabaldón T. (2009). trimAl: A tool for automated alignment trimming in large-scale phylogenetic analyses. Bioinformatics.

[55] Minh B.Q., Schmidt H.A., Chernomor O., Schrempf D., Woodhams M.D., von Haeseler A., Lanfear R. (2020). IQ-TREE 2: New Models and Efficient Methods for Phylogenetic Inference in the Genomic Era. Mol. Biol. Evol..

[56] Huelsenbeck J.P., Ronquist F. (2001). MRBAYES: Bayesian inference of phylogenetic trees. Bioinformatics.

[57] Mirarab S., Warnow T. (2015). ASTRAL-II: Coalescent-based species tree estimation with many hundreds of taxa and thousands of genes. Bioinformatics.

[58] Smith S.A., Moore M.J., Brown J.W., Yang Y. (2015). Analysis of phylogenomic datasets reveals conflict, concordance, and gene duplications with examples from animals and plants. BMC Evol. Biol..

[59] Yang Z. (2007). PAML 4: Phylogenetic analysis by maximum likelihood. Mol. Biol. Evol..

[60] Choi H.-I., Kim N.-H., Lee J., Choi B.S., Kim K.D., Park J.Y., Lee S.-C., Yang T.J. (2013). Evolutionary relationship of *Panax ginseng* and *Panax quinquefolius* inferred from sequencing and comparative analysis of expressed sequence tags. Genet. Resour. Crop Evol..

[61] Xie J.M., Chen Y.R., Cai G.J., Cai R.L., Hu Z., Wang H. (2023). Tree Visualization by One Table (tvBOT): A web application for visualizing, modifying and annotating phylogenetic trees. Nucleic Acids Res..

[62] Huson D.H., Scornavacca C. (2012). Dendroscope 3: An Interactive Tool for Rooted Phylogenetic Trees and Networks. Syst. Biol..

[63] Wang N., Yang Y., Moore M.J., Brockington S.F., Walker J.F., Brown J.W., Liang B., Feng T., Edwards C., Mikenas J. (2019). Evolution of Portulacineae Marked by Gene Tree Conflict and Gene Family Expansion Associated with Adaptation to Harsh Environments. Mol. Biol. Evol..

[64] Jiang Z.Q., Tu L.C., Yang W.F., Zhang Y.F., Hu T.Y., Ma B.W., Lu Y., Cui X.M., Gao J., Wu X.Y. (2021). The chromosome-level reference genome assembly for *Panax notoginseng* and insights into ginsenoside biosynthesis. Plant Commun..

[65] Kim D., Landmead B., Salzberg S.L. (2015). HISAT: A fast spliced aligner with low memory requirements. Nat. Methods.

[66] Danecek P., Bonfield J.K., Liddle J., Marshall J., Ohan V., Pollard M.O., Whitwham A., Keane T., McCarthy S.A., Davies R.M. (2021). Twelve years of SAMtools and BCFtools. Gigascience.

[67] Liao Y., Smyth G.K., Shi W. (2014). featureCounts: An efficient general purpose program for assigning sequence reads to genomic features. Bioinformatics.

[68] Eddy S.R. (1998). Profile hidden Markov models. Bioinformatics.

[69] Sayers E.W., Beck J., Bolton E.E., Brister J.R., Chan J., Comeau D.C., Connor R., DiCuccio M., Farrell C.M., Feldgarden M. (2024). Database resources of the National Center for Biotechnology Information. Nucleic Acids Res..

[70] Wang J.Y., Chitsaz F., Derbyshire M.K., Gonzales N.R., Gwadz M., Lu S.N., Marchler G.H., Song J.S., Thanki N., Yamashita R.A. (2023). The conserved domain database in 2023. Nucleic Acids Res..

